# What Makes You Can Also Break You, Part II: Mitochondrial Permeability Transition Pore Formation by Dimers of the F_1_F_O_ ATP-Synthase?

**DOI:** 10.3389/fonc.2013.00140

**Published:** 2013-05-30

**Authors:** Gyorgy Szabadkai, Christos Chinopoulos

**Affiliations:** ^1^Department of Cell and Developmental Biology, Consortium for Mitochondrial Research, University College LondonLondon, UK; ^2^Department of Biomedical Sciences, University of PaduaPadua, Italy; ^3^Department of Medical Biochemistry, Semmelweis UniversityBudapest, Hungary

Only a few months ago we commented on the findings by Bonora et al. (2013), regarding the role of the c ring of the F_1_F_O_ ATP synthase forming the mitochondrial permeability transition (mPT). We forecasted that this is just the opening of yet another shake up in the mitochondrial field, and more studies on the topic will soon follow (Chinopoulos and Szabadkai, 2013). Indeed, now a paper by Giorgio et al. (2013) proposes that the mPT is shaped up by dimers of whole F_1_F_O_ ATP-synthase complexes.

The deleterious effect of the mPT on cellular metabolism and survival is a fundamental target in cellular pathology, but the lack of knowledge on its molecular identity largely excluded the possibility to exploit its central position in cell death induction. Following a long debate of the presumed components (see Chinopoulos and Szabadkai, 2013 and references therein) cyclophilin D (cypD) emerged as an essential regulator and thus a *bona fide* component associated with the actual pore. Successively, the group of Bernardi and others (Giorgio et al., 2009; Chinopoulos et al., 2011) have revealed that cypD binds and regulates the F_1_F_O_ ATP-synthase. Lastly, the present work characterizes the cypD – F_1_F_O_ ATP-synthase interaction from the point of view of mPT formation, providing evidence that cypD targets the OSCP subunit of the lateral stalk. Moreover, in addition to presenting a series of biochemical and functional evidence indicating that the mPT is correlated with the cypD-OSCP interaction, they directly addressed the pore forming ability of purified F_1_F_O_ ATP-synthases. It is well established that the mPT represents the opening of a high-conductance channel, called the “mitochondrial megachannel” (MMC), identified by patch-clamping the inner membrane (Kinnally et al., 1989; Petronilli et al., 1989; Szabó et al., 1992; De Marchi et al., 2006). Intriguingly, now Giorgio et al. (2013) show that by incorporating purified F_1_F_O_ ATP-synthase dimers in liposomes, electrophysiological recordings identical to the MMC can be obtained. Whilst these results provide the most direct evidence so far for mPT pore formation by the F_1_F_O_ ATP-synthase, again, they raise a series of new questions.

First, in order to obtain a reversible ion permeable pore at the dimerization interface of a membrane protein dimer, two hydrophobic surfaces should be able to provide a hydrophilic lining while the pore is assembled in order to allow ion flow. Then, when the channel is inactivated, these surfaces should become hydrophobic again when the dimer is disassembled, to allow interaction with membrane lipids. In theory, this could be achieved by rotation of amphipathic alpha-helices. If the interpretations of Giorgio et al. are correct, such a helix should be present at the surface of the F_1_F_O_ ATP-synthase, precisely at the site where two ATP-synthases interact to form the dimers. Unfortunately, there is insufficient knowledge about the structure of the F_O_ portion to validate this possibility. In addition, in spite of the enormous diversity of known gating mechanisms of channels found on the plasma membrane and intracellular organelles, a common denominator is that there is no phospholipid bilayer present in the pore region. Yet, Giorgio et al. showed that the MMC electrophysiological signature can be reproduced by incorporating purified F_1_F_O_ ATP-synthase dimers in liposomes, where phospholipids are present within and in between the dimers. We can envisage three possibilities to explain their findings: (1) there is channel gating in the presence of an imposing phospholipid bilayer. The concept of a lipidic pore, induced by dimerization of proteins has been proposed previously (Tait and Green, 2010), however, this pore could not exhibit rapid gating properties, which is characteristic of the MMC. (2) An additional protein forms the true pore of the mPT, which is present only in the F_1_F_O_ ATP-synthase dimeric complexes. This might explain why they have obtained functional pores when reconstituting dimers and not monomers. Relevant to this possibility, the inhibitory factor 1 (IF1) is being increasingly recognized to promote dimerization of the mitochondrial F_1_F_O_ ATP-synthase (García et al., 2006; Campanella et al., 2008), thus it would be interesting to see if IF1 has any role in mPT formation. (3) The dimerization of the mitochondrial F_1_F_O_ ATP-synthase distorts either c ring to the point of conferring properties of the MMC. As highlighted in our previous commentary, this idea is not far-fetched (Chinopoulos and Szabadkai, 2013), and would consolidate the findings of Bonora et al. (2013) with the model of Giorgio et al. (2013). Indeed, in their electrophysiological recordings, both F_1_F_O_ ATP-synthases forming a dimer must have been present within the patched area, thus their results show that dimerization is required for MMC activity, but not that the actual pore forms in between the dimers.

Second, Bernardi's group has previously shown that cells depleted of mitochondrial DNA (rho_0_ cells) still exhibit mPT (Masgras et al., 2012). The rho_0_ cells express truncated ATP-synthase monomers since they lack the mitochondrially encoded subunits a and A6L (Carrozzo et al., 2006). In the absence of these subunits, rho_0_ cells can still form dimeric ATP-synthase structures (mediated by interactions of other subunits) although at considerably reduced amounts due to structural instability of the oligomers (Wittig et al., 2010). However, the very same subunits are critical for the model suggesting mPT formation by ATP-synthase dimers (Bernardi, 2013). Thus the redundancy of subunits a and A6L for mPT in rho_0_ cells but their necessity for the ATP-synthase dimers formation will require some further clarification.

Third, the results by Giorgio et al. (2013) suggest that the activity of the F_1_F_O_ ATP synthase has a strong impact on Ca^2+^ induced mPT. They show that when the complex works in the “reverse” ATP hydrolysis mode, it is far less sensitive to Ca^2+^ as compared to when it functions in the “forward” ATP synthesis mode. Dimerization of the F_1_F_O_ ATP-synthase complex has been shown to be associated with increased ATP-synthetic efficiency, again probably driven by associated proteins such as IF1 (Campanella et al., 2008), but this state is usually coupled to increased cell survival, not compatible with mPT. Thus, integrating the pro-death and pro-survival functions in one molecular complex does not seem straightforward.

Nonetheless, now that there is an apparently solid clue regarding the molecular identity of the mPT, a vast amount of functional data that has accumulated in the past few decades of research (reviewed, e.g., in Rasola and Bernardi, 2007; Chinopoulos and Adam-Vizi, 2012) can be re-addressed in light of the scheme proposed by Giorgio et al. (2013). Most of them will appeal to structural biologists: do the histidyl residues mediating the effect of matrix pH on the opening probability of mPT reside on the F_1_F_O_ ATP-synthase dimers? Where are the redox-sensitive sites (modulated by either matrix pyridine nucleotides or through vicinal thiols depending on GSH pools) which are affected by electron flux and surface potential that probably comprise the voltage-sensor of the mPT? Can they be located on the F_1_F_O_ ATP-synthase dimers, or do they reside on an interacting protein? Are the critical arginine residues that modulate the voltage dependence and the opening-closing mechanism of the pore, intrinsic to the dimers? Are the F_1_F_O_ ATP-synthase dimers direct targets of amphipathic anions that favor pore opening, or of polycations, amphipathic cations, and positively charged peptides that inhibit the pore? What is the connection between the established role of electron flow through the respiratory chain complex I in pore opening (Fontaine et al., 1998) and the F_1_F_O_ ATP-synthase dimers? These and probably a plethora of other questions will likely keep occupied many investigators in the field.

Finally, it will be interesting, but equally challenging to test the validity of this mPT model not just in liposomes but in a living system, probably by using genetic models with modified expression of subunits required for higher order complex formation. Such studies would certainly clarify the pathophysiological relevance of the findings by Giorgio et al. (2013) and set the future directions of this research field, which now has been definitely revived.
